# Light-Activated Heterostructured Nanomaterials for Antibacterial Applications

**DOI:** 10.3390/nano10040643

**Published:** 2020-03-30

**Authors:** Chinmaya Mutalik, Di-Yan Wang, Dyah Ika Krisnawati, Achmad Jazidie, Sibidou Yougbare, Tsung-Rong Kuo

**Affiliations:** 1 International Ph.D. Program in Biomedical Engineering, College of Biomedical Engineering, Taipei Medical University, Taipei 11031, Taiwan; cmutalik41@gmail.com (C.M.); d845107003@tmu.edu.tw (S.Y.); 2 Department of Chemistry, Tunghai University, Taichung 40704, Taiwan; diyanwang@thu.edu.tw; 3 Center for Science and Technology, Tunghai University, Taichung 40704, Taiwan; 4 Dharma Husada Nursing Academy, Kediri, East Java 64114, Indonesia; dyahkrisna77@gmail.com; 5 Department of Electrical Engineering, Institut Teknologi Sepuluh Nopember, Surabaya 60111, Indonesia; rektor@unusa.ac.id; 6 University Nahdlatul Ulama Surabaya, Surabaya 60111, Indonesia; 7 Graduate Institute of Nanomedicine and Medical Engineering, College of Biomedical Engineering, Taipei Medical University, Taipei 11031, Taiwan

**Keywords:** heterostructured nanomaterial, antibacterial agent, antibacterial mechanism, reactive oxygen species, light-activated, synergistic effect

## Abstract

An outbreak of a bacterial contagion is a critical threat for human health worldwide. Recently, light-activated heterostructured nanomaterials (LAHNs) have shown potential as antibacterial agents, owing to their unique structural and optical properties. Many investigations have revealed that heterostructured nanomaterials are potential antibacterial agents under light irradiation. In this review, we summarize recent developments of light-activated antibacterial agents using heterostructured nanomaterials and specifically categorized those agents based on their various light harvesters. The detailed antibacterial mechanisms are also addressed. With the achievements of LAHNs as antibacterial agents, we further discuss the challenges and opportunities for their future clinical applications.

## 1. Introduction

Antibacterial studies have become more significant because of the increased of multidrug-resistant (MDR) bacteria in this era [1,2,3,4,5,6,7,8,9,10]. Various mechanisms can stimulate antibacterial resistance, including overuse of antibiotics and the delivery of bacteria by different routes [11,12,13]. A recent report indicates that by 2050, antibacterial resistance will be a tremendous public health threat with the potential to cause the death of ten million people each year [14]. Therefore, development of new types of antibacterial agents against MDR bacteria is an urgent task. The development of new antibacterial agents inspired the researchers for the investigation of nanomaterials which can eliminate MDR bacteria without the help of antibiotics [15,16,17,18,19].

Nanomaterials—including metals, metal oxide, semiconductors and polymers—have been extensively studied for applications in nanoscience and nanotechnology, due to their superior chemical and physical properties [20,21,22,23,24,25,26,27,28,29,30,31,32,33,34,35]. Among nanomaterials, the heterostructured nanomaterials have shown unique optical properties, including the increase of light absorption and the extension of absorption region [36,37,38,39,40,41]. Recently, great advancements have been reported in the application of LAHNs as antibacterial agents. With the rise of light absorption and the extension of absorption region, heterostructured nanomaterials have shown superior antibacterial activity, based on reactive oxygen species (ROS) generation under light illumination [42,43,44,45,46,47,48]. The emphasis of this review is on the mechanism study of light-induced ROS generation from heterostructured nanomaterials by synergistic effect. Recent achievements with the uses of LAHNs as antibacterial agents are classified by their various light harvesters. Finally, challenges and perspectives for LAHNs as antibacterial agents are also provided.

## 2. Antibacterial Nanomaterials Based on Light-induced ROS Generation

**Figure 1:**
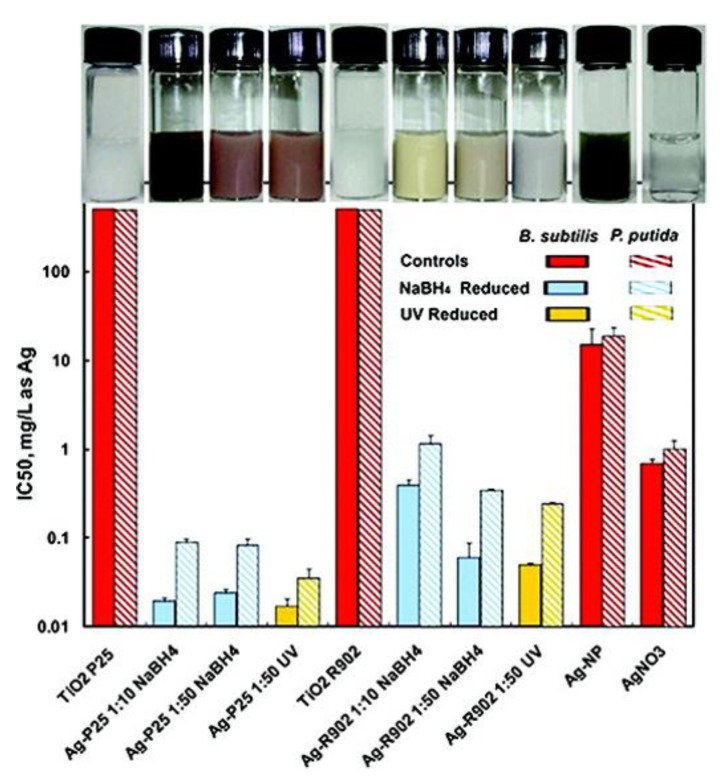
IC50 values of different hybrid Ag-TiO_2_ nanoparticles with and without UV light irradiations. TiO_2_ P25 (Degussa P25) and TiO_2_ R-902 (DuPont Ti-Pure R-902) were two commercial TiO_2_ nanoparticles. Two different molar ratios of AgNO_3_ and TiO_2_ including 1:10 and 1:50 were respectively employed to prepare Ag nanoparticles deposited onto the TiO_2_ nanoparticles by NaBH_4_ reduction and UV photoreduction to form the samples of Ag-P25 1:10 NaBH_4_, Ag-P25 1:50 NaBH_4_, Ag-P25 1:50 UV, Ag-R902 1:10 NaBH_4_, Ag-R902 1:50 NaBH_4_ and Ag-R902 1:50 UV. Ag nanoparticles and AgNO_3_ were purchased from QuantumSphere, Inc. Reproduced with permission from ref. [68]. Copyright © 2011, American Chemical Society.

Titanium dioxide (TiO_2_) materials have been found to possess remarkable biocompatibility and low cell toxicity with significant antibacterial activity [49,50,51,52,53,54,55,56,57,58]. Recently, TiO_2_-based nanomaterials were popular materials with substantial amount of antibacterial activities, and their antibacterial activities could be further enhanced by light irradiations with various wavelengths, including ultraviolet (UV) light, visible light and near-infrared (NIR) light due to the increase of ROS generation [59,60,61,62,63,64,65,66,67]. For example, with UV light irradiation, heterostructured Ag-TiO_2_ nanoparticles have displayed higher bactericidal activity compared to that of only UV irradiation, Ag nanoparticles under UV irradiation or TiO_2_ nanoparticles under UV irradiation (Figure 1) [68]. With UV light illumination, the augmentation of bactericidal activity of hybrid Ag-TiO_2_ nanoparticles indicated that Ag nanoparticles loaded onto TiO_2_ nanoparticles served as electron traps to prevent recombination of electron and hole in hybrid Ag-TiO_2_ nanoparticles. The photo-exited electrons were generated and then transferred to Ag nanoparticles to extend the life of electron-hole pairs. In the system of hybrid Ag-TiO_2_ nanoparticles, the increases of electron-hole pairs enhanced ROS generation for the destruction of bacterial membrane or DNA [69,70,71,72,73,74]. In the work, hybrid Ag-TiO_2_ nanoparticles provided more effective bactericides with light irradiations; however, the uses of hybrid Ag-TiO_2_ nanoparticles as an antibacterial agent were challenging in practical application due to their high cytotoxicity and facile accumulation in tissues and organs.

**Figure 2:**
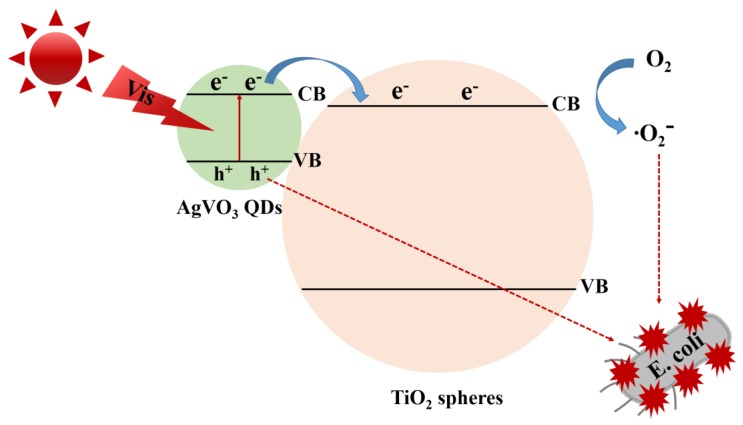
Antibacterial mechanism of TiO_2_/AgVO_3_ nanocomposites with visible light illumination. Reproduced with permission from ref. [75]. Copyright © 2019, Elsevier.

Nanocomposites of AgVO_3_ quantum dots (QDs) deposited onto TiO_2_ nanospheres (TiO_2_/AgVO_3_) have shown high-performance photocatalytic capability with visible light irradiation [75,76,77]. TiO_2_/AgVO_3_ nanocomposites incubated with *E. coli* have been investigated to study their light-induced bacterial inactivation with illumination of visible light. In the control experiment, after visible light irradiation over 120 min, TiO_2_ nanospheres were inactivated only 0.13 log of *E. coli*. With the use of TiO_2_/AgVO_3_ nanocomposites as photocatalysts, all of *E. coli* were killed under visible light irradiation over 120 min. After TiO_2_/AgVO_3_ nanocomposites incubated with *E. coli*, with visible light irradiation; the shape of *E. coli* was destroyed. Moreover, there were no obvious changes of bacterial inactivity of TiO_2_/AgVO_3_ nanocomposites after three cycling antibacterial tests under visible light irradiation indicating that TiO_2_/AgVO_3_ nanocomposites were good photocatalysts with superior stability. For the photocatalytic disinfection mechanism, AgVO_3_ QDs were excited to offer light-induced pairs of electrons and holes with visible light illumination and then the light-induced electrons located in the conduction band of AgVO_3_ QDs were easily delivered to the conduction band of TiO_2_ (Figure 2). The light-induced electrons in the conduction band of TiO_2_ could react with oxygen in medium to form ⋅O_2_^−^. Moreover, the holes located in the valence band of AgVO_3_ QDs could be delivered to their surface to inhibit the growth of *E. coli*. To sum up, the light-induced electron-hole pairs in TiO_2_/AgVO_3_ nanocomposites were efficiently separated to improve the light-induce antibacterial activity of *E. coli*.

**Figure 3:**
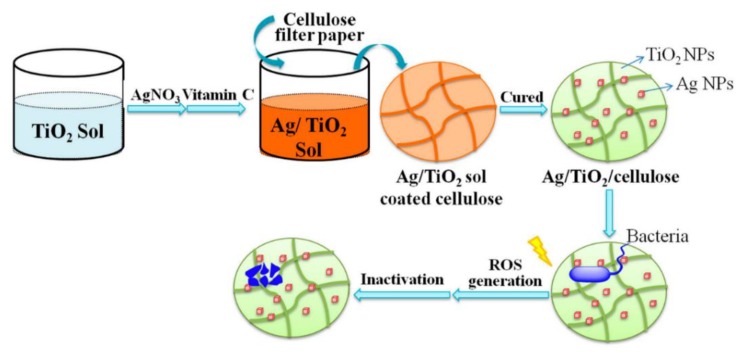
Schematic illustration of preparation of the nanocomposite film of Ag/TiO_2_/cellulose and its antibacterial mechanism. Reproduced with permission from ref. [81]. Copyright © 2018, MDPI.

Natural polymers of cellulose are intensively used in daily life [78,79,80]. However, the application of cellulose has been limited, due to its susceptibility to microorganism growth. In recent advancements, a simple sol-gel approach has been used to conjugate the cellulose scaffold with Ag/TiO_2_ nanoparticles (Ag/TiO_2_/cellulose) against bacteria (Figure 3) [81]. The antibacterial activities of nanocomposite film of Ag/TiO_2_/cellulose, film of pristine cellulose and nanocomposite film of TiO_2_/cellulose have been investigated with *E. coli* with and without UV light irradiation. The bactericidal performance of the nanocomposite film of Ag/TiO_2_/cellulose with Ag content of 0.030 wt% has shown the best 99.9% inactivation of *E. coli* with UV light illumination. The results suggest that the nanocomposite film of Ag/TiO_2_/cellulose exhibit superior bactericidal performance against *E. coli* because of the synergistic effect of Ag nanoparticles and anatase TiO_2_ nanoparticles. Under UV light irradiation, TiO_2_ nanoparticles may generate ROS—including ⋅OH, ⋅O_2_^−^ and H_2_O_2_—prompting bacterial death. Moreover, Ag nanoparticles may capture electrons, restraining the recombination of photon-induced electron/hole pairs for the increase of ROS formation. Overall, the bactericidal performance of the nanocomposite film of Ag/TiO_2_/cellulose was significantly enhanced under UV light irradiation.

**Figure 4:**
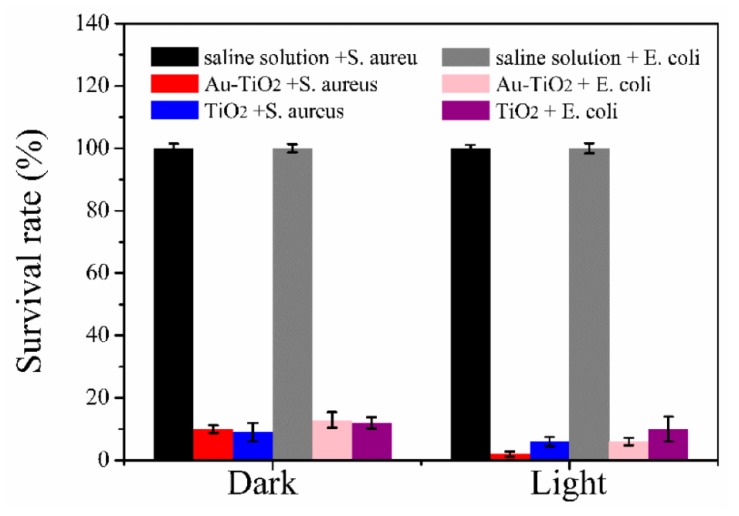
Antibacterial activities of saline solution, sodium alginate/TiO_2_ nanocomposite (ST) film and sodium alginate/Au-TiO_2_ nanocomposite (SAT) film for *S. aureus* and *E. coli* with and without visible light illumination, respectively. Reproduced with permission from ref. [82]. Copyright © 2018, MDPI.

Au-TiO_2_ nanocomposites embedded into a degradable and antibacterial sodium alginate films have been developed by food packaging industries against bacteria [82]. Sodium alginate/Au-TiO_2_ nanocomposite (SAT) films were enabled to absorb light form UV to visible wavelength and to improve their hydrophilicity and shape stability. As shown in Figure 4, both sodium alginate/TiO_2_ nanocomposite (ST) films and SAT films exhibit distinct antibacterial activities for *S. aureus* and *E. coli* in dark. With the use of SAT films, 90% of *S. aureus* and 97.1% *E. coli* were killed without light illumination, respectively. However, after light illumination for 20 min, the antibacterial abilities of SAT films were respectively improved ~60% and ~50% for *S. aureus* and *E. coli*. The improvement of antibacterial abilities of SAT films may be attributed to that the nanocomposites of Au-TiO_2_ increased light absorption and transfer capability due to their plasmonic effect. The plasmonic Au nanoparticles in SAT films may harvest light to produce light-induced photons for the increase of ROS to kill bacteria.

**Figure 5:**
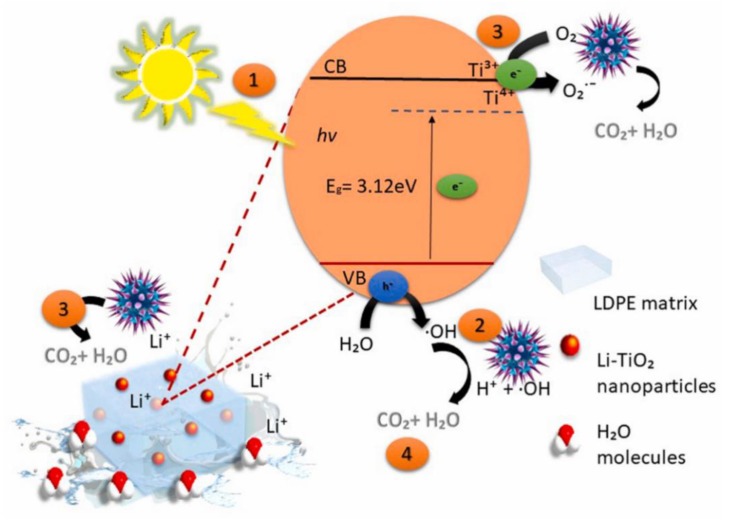
The antimicrobial mechanism of the heterostructured nanocomposites of Li-TiO_2_/LDPE against *S. aureus*. Abbreviation: valence band: VB; conduction band: CB. Reproduced with permission from ref. [83]. Copyright © 2019, Elsevier.

The nanocomposites of lithium-titanate (Li-TiO_2_) in the low-density polyethylene (LDPE) matrix have shown a significant increase in killing efficiency for *S. aureus* with visible light illumination [83]. In the heterostructured nanocomposites of Li-TiO_2_/LDPE, the oxygen vacancies of Ti^3+^ and interaction of Li-O-Ti bond reduced the band gap of TiO_2_ nanoparticles, resulting in their response to visible light (Figure 5). LDPE alone did not show any antibacterial activity for *S. aureus*. With the dopant of Li-TiO_2_ of 1 wt%, the heterostructured nanocomposites of Li-TiO_2_/LDPE inhibited the growth of *S. aureus* by 94% after visible light irradiation for 6 h, then raised the inhibition rate to 99% within 12 h under visible light irradiation. From the results of the scavenger test, the intensified bactericidal effect of the heterostructured nanocomposites of Li-TiO_2_/LDPE were ascribed to the productions of powerful oxidants including ⋅OH and ⋅O_2_^−^ under visible light irradiation. These powerful oxidants may particularly react with the polyunsaturated phospholipid composition of the bacterial membrane to form water and carbon dioxide.

**Figure 6:**
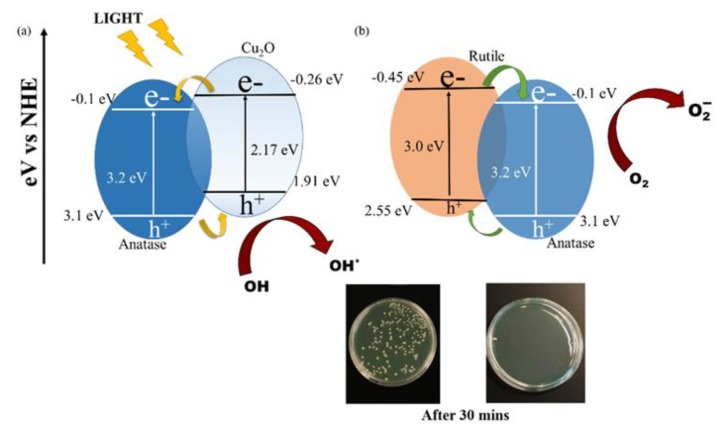
Mechanisms of photocatalytic antibacterial activity (**a**) p-n junction by heterostructured Cu_2_O and TiO_2_ (**b**) Type-2 heterostructures of anatase and rutile TiO_2_ nanoparticles. Images of *S. aureus* colonies in the agar plates with (right) and without (left) Cu-TiO_2_ nanomaterials after 30 min illumination. Reproduced with permission from ref. [84]. Copyright © 2018, MDPI.

The heterostructured nanomaterials of copper-doped TiO_2_ (Cu-TiO_2_) have been applied as an antibacterial agent against *E. coli* and *S. aureus* with visible light illumination [84]. By utilizing dopant of oxidizing copper, Cu-TiO_2_ nanomaterials revealed the increase of absorption in visible region and the band gap of Cu-TiO_2_ nanomaterials was reduced to 2.8 eV. The photo-induced bactericidal activity of Cu-TiO_2_ heterostructured nanomaterial have been respectively executed with and without visible light illumination (Figure 6). Without photocatalysts, the *E. coli* and *S. aureus* bacteria continued growing under light and dark conditions. Moreover, in the dark, the Cu-TiO_2_ nanomaterials, anatase TiO_2_ nanoparticles and rutile TiO_2_ nanoparticles had no significant bacterial growth after culture for 90 min. After visible light irradiation for 30 min, the Cu-TiO_2_ nanomaterials had achieved 99.9999% bacterial reduction. However, to obtain 99.9999% bacterial reduction, both anatase and rutile TiO_2_ nanoparticles required light irradiation for 60 and 90 min, respectively. In comparison with anatase and rutile TiO_2_ nanoparticles, Cu-TiO_2_ nanomaterials showed better antibacterial activity. The results indicate that the dopant of Cu in Cu-TiO_2_ nanomaterials may improve the visible absorption efficiency to enhance bacterial inactivation. After the absorption of light, the charge carriers formed on p-n junction of Cu_2_O-TiO_2_ nanomaterials and then reacted with the oxygen and water incorporated on the surface of Cu_2_O-TiO_2_ nanomaterials to become radicals to disrupt bacterial membrane and cause bacterial gene alteration to kill bacteria.

**Figure 7:**
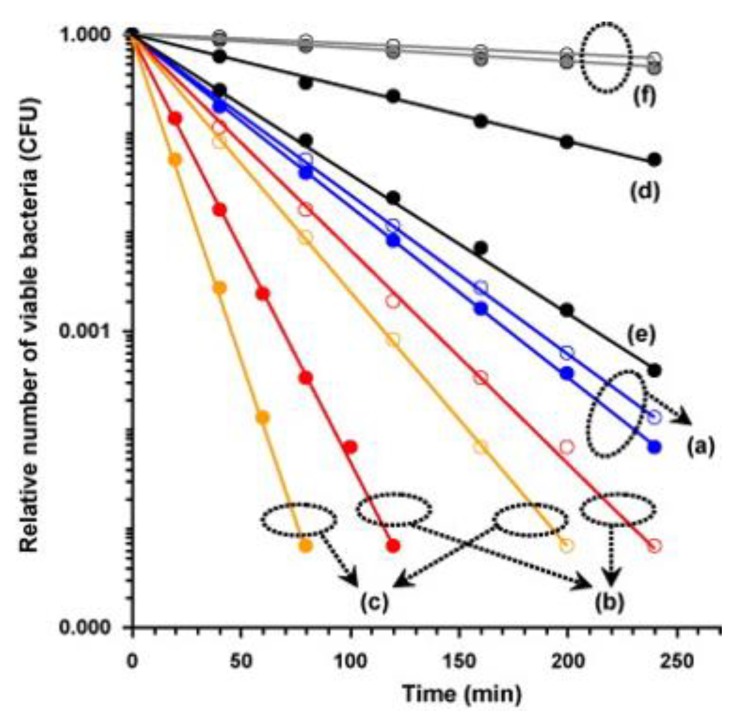
Relative number of viable *E. coli* on surface of (**a**) the anatase TiO_2_ (**b**) the α-Fe_2_O_3_ and (**c**) the TiO_2_/Fe_2_O_3_ thin films under visible light irradiation. (**d**) The α-Fe_2_O_3_ and (**e**) the TiO_2_/Fe_2_O_3_ thin films without light illumination. (**f**) Control samples containing the solution without H_2_O_2_ (○) and with H_2_O_2_ (●) with light illumination. The black circles indicated the samples of a, b, c and f containing the solution without H_2_O_2_ (○) and with H_2_O_2_ (●) with light illumination. Reproduced with permission from ref. [85]. Copyright © 2009, Elsevier.

The thin film of photocatalyst of α-Fe_2_O_3_ nanograin chains incorporated with anatase TiO_2_ nanolayer (TiO_2_/Fe_2_O_3_) exhibited bactericidal activity against *E. coli* with visible light illumination [85]. The bacterial inactivation of the anatase TiO_2_, α-Fe_2_O_3_ and TiO_2_/α-Fe_2_O_3_ thin films has been studied in the bacterial model of *E. coli* under visible light illumination, with and without H_2_O_2_ (Figure 7). As shown in Figure 7, TiO_2_/α-Fe_2_O_3_ thin film reveals the reduction rate of 2.6 × 10^−2^ and 6.5 × 10^−2^ min^−1^ without and with H_2_O_2_ for *E. coil*, reflecting a better antibacterial activity than anatase TiO_2_ and bare α-Fe_2_O_3_ thin film. The better bacterial inactivity of TiO_2_/α-Fe_2_O_3_ thin film may be ascribed to the heterostructure of TiO_2_/Fe_2_O_3_. Through visible light irradiation onto TiO_2_/Fe_2_O_3_ heterojunction, electrons located in the valence band of Fe_2_O_3_ may be excited to the conduction band—and then, electrons located in the valence band of TiO_2_ may be driven into the valance band of Fe_2_O_3_. Therefore, the anatase TiO_2_ nanolayer coated with TiO_2_/α-Fe_2_O_3_ thin film could be applied to enhance of the photocatalytic capability of the coated Fe_2_O_3_ nanograins. The anatase TiO_2_ nanolayer could increase the production of Fe^2+^ to react with the H_2_O_2_ for the generation of OH radicals resulted from the reactions of Fenton and photo-Fenton. Furthermore, water or hydroxyl groups adsorbed onto anatase TiO_2_ nanolayer may be used to trap the holes to produce ROS of hydroxyl radicals for the bactericidal activity enhancement to bacteria.

**Figure 8:**
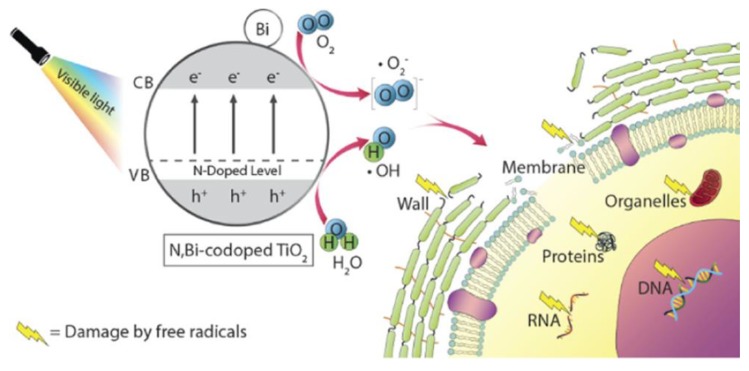
Schematic illustration of light-induced antibacterial mechanism of N, Bi-codoped TiO_2_ photocatalysts. Bacterial DNA should locate in cytoplasm. Reproduced with permission from ref. [86]. Copyright © 2019, American Chemical Society.

Recent developments of implants have focused on the design of their surface in terms of bacterial inactivation and reusable feasibility. The dopants nitrogen (N) and bismuth (Bi) in TiO_2_— incorporated with the plasma electrolytic oxidation (PEO)—revealed light-induced antibacterial activity and re-activation potential [86]. With the dopant Bi, the band gap energy of TiO_2_ was shifted to the visible light region. To confirm the in vitro antibacterial effect, the photocatalysts of TiO_2_ coated with urea (Urea-TiO_2_ group), TiO_2_ doped with Bi (Bi-TiO_2_ group) and TiO_2_ co-doped with urea and Bi (Urea, Bi-TiO_2_ group) were applied as antibacterial agents for two biofilms, including *Streptococcus sanguinis* and *Actinomyces naeslundii*. The results of the in vitro antibacterial tests indicated that the urea, Bi-TiO_2_ group presented the best performance in bactericidal activity with visible light illumination. With a synergistic effect of N and Bi under visible light illumination, the narrowed band gap of Bi_2_O_3_ combined with TiO_2_ could facilitate electrons migration from the valence band to the conduction band located in TiO_2_ and Ti^3+^ sites to react with O_2_ to form ⋅O_2_^−^ and also ⋅OH radical could be produced according to the reaction of holes located in the valence band of TiO_2_ with H_2_O (Figure 8). The ROS of ⋅O_2_^−^ and ⋅OH may induce damage of bacterial wall, membrane, organelles, proteins and genetic materials (DNA, RNA) to cause death of bacteria.

**Figure 9:**
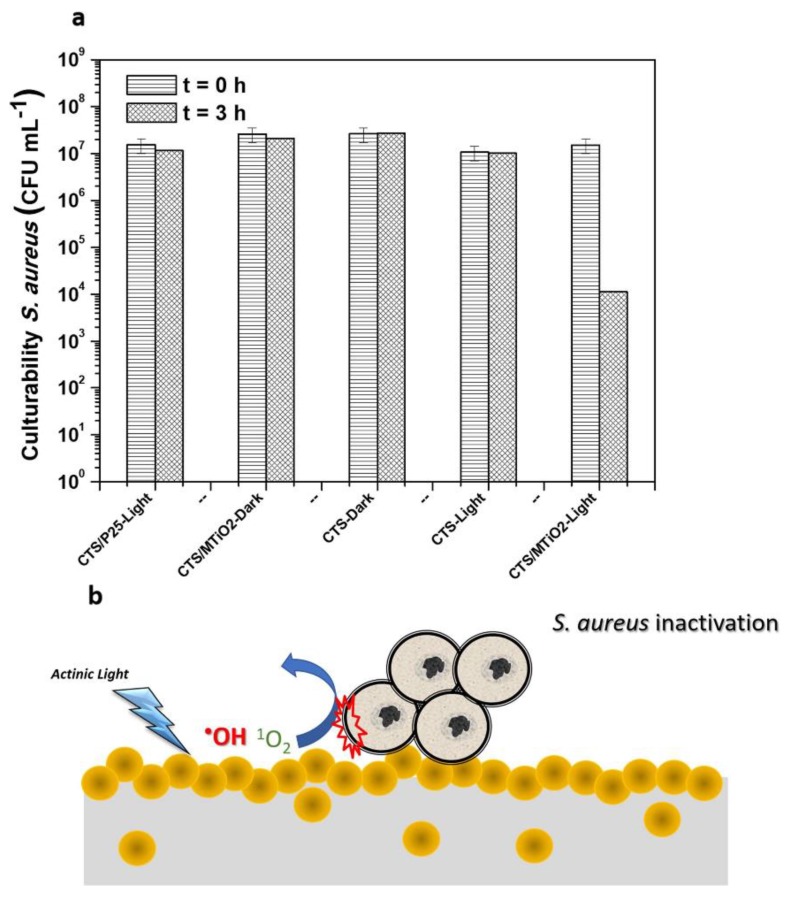
(**a**) Bacterial culturability of *S. aureus* with and without actinic light irradiation. (**b**) Antibacterial mechanism of CTS/MTiO_2_ films under actinic light irradiation. Reproduced with permission from ref. [87]. Copyright © 2019, Elsevier.

In recent times, nanocomposites of chitosan films containing melon/TiO_2_ (CTS/MTiO_2_) have been applied for self-cleaning of malachite green and light-induced antibacterial surfaces of *S. aureus* under light irradiation [87]. As the most common graphitic carbon nitride material (g-C_3_N_4_), melon is a visible-light harvesting molecule. The bacterial inactivation of CTS/MTiO_2_ nanocomposites was assessed against *S. aureus* with light illumination (Figure 9). After incubated with *S. aureus* for 3 h, CTS/P25, CTS/MTiO_2_ and CTS have revealed different antibacterial activities with and without actinic light irradiations as shown in Figure 9a. Furthermore, in Figure 9b, antibacterial mechanism of CTS/MTiO_2_ films has been explained under actinic light irradiation. For the control experiments, chitosan and CTS/P25 films showed no significant bacterial inactivation with and without actinic light irradiation for 3 h. On the other hand, CTS/MTiO_2_ films exhibited an antibacterial rate 99% of *S. aureus* after 3 h of actinic light irradiation. Previous studies indicated that chitosan films combined neat TiO_2_ nanoparticles exhibited a bactericidal efficiency of 99.9% for *E. coli* under light irradiation for 4 h. However, under actinic light irradiation for 4 h, chitosan films combined neat TiO_2_ nanoparticles had no antibacterial activity for *S. aureus*. In this aspect, CTS/MTiO_2_ films had superior antibacterial activity for *S. aureus*. For the antibacterial mechanism, CTS/MTiO_2_ films were induced for the formation of ROS such as ⋅OH, ^1^O_2_, ⋅O_2_^−^ and H_2_O_2_ to damage bacterial cell membrane with actinic light irradiation.

**Figure 10:**
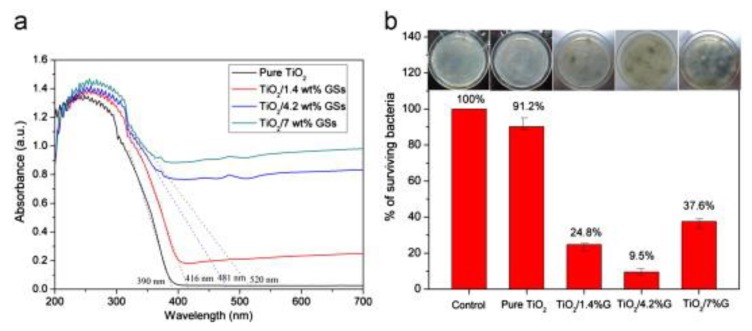
(**a**) UV-Vis absorption spectra of pure TiO_2_ nanoparticles, TiO_2_/GSs with TiO_2_/1.4 wt%, TiO_2_/GSs with TiO_2_/4.2 wt% and TiO_2_/GSs with TiO_2_/7 wt%. (**b**) Viabilities of *E. coli* incubated with various TiO_2_-based nanocomposites under visible light illumination for 12 h. The insets are the corresponding photographs of agar plates. Reproduced with permission from ref. [88]. Copyright © 2013, Elsevier.

Nanocomposites of uniform TiO_2_ nanoparticles and graphene sheets (TiO_2_/GSs) have been fabricated via a facile redox reaction for antibacterial applications [88,89,90,91,92,93]. TiO_2_ nanoparticles were anchored on the surfaces of GSs by chemical bonds. Moreover, nanocomposites of TiO_2_/GSs exhibited broad absorption region from UV light to visible light. The photocatalysts of TiO_2_/GSs were used to kill *E. coli* under visible light illumination. Uniform TiO_2_ nanoparticles showed lower antibacterial capability in comparison with TiO_2_/GSs nanocomposites due to their large band gap, resulting in very low light absorption in the visible region (Figure 10). UV-Vis absorption spectra of pure TiO_2_ nanoparticles, TiO_2_/GSs with TiO_2_/1.4 wt%, TiO_2_/GSs with TiO_2_/4.2 wt% and TiO_2_/GSs with TiO_2_/7 wt% have shown in Figure 10a. Under visible light illumination for 12 h, viabilities of *E. coli* incubated with various TiO_2_-based nanocomposites have been respectively calculated as shown in Figure 10b. Under visible light illumination, the nanocomposites of TiO_2_/GSs with TiO_2_/4.2 wt% revealed the best antibacterial activity in comparison with TiO_2_/GSs with various weight ratios, including TiO_2_/1.4 wt% and TiO_2_/7 wt%. The superior antibacterial activity of TiO_2_/GSs nanocomposites may be explained by the increase of light absorption in visible region and effective separation of photo-generated electron-hole pairs because graphene could be applied as an electron acceptor and transporter. With the effective separation of light-generated pairs of electrons and holes, TiO_2_/GSs nanocomposites may produce more ROS, including ⋅OH and ⋅O_2_^−^ for disinfection activity of *E. coli*. For the nanocomposites of TiO_2_/GSs with TiO_2_/7 wt%, the decrease of antibacterial activity may be ascribed to that the active TiO_2_ nanoparticles were covered by a large number of GSs, forming a shield of active sites on TiO_2_ nanoparticles.

**Figure 11:**
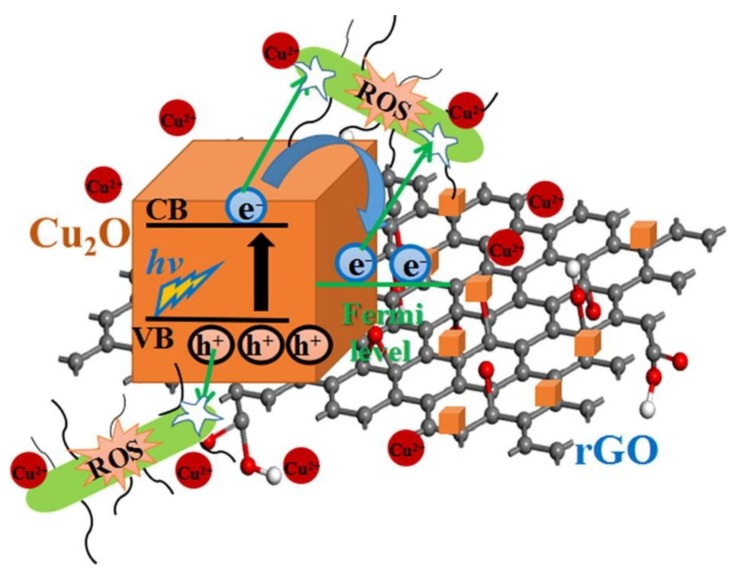
Reactive oxygen species (ROS) production caused by the heterostructured nanocomposites rGO-Cu_2_O under light illumination. Reproduced with permission from ref. [94]. Copyright © 2019, Elsevier.

Heterostructured nanocomposites of reduced graphene oxide and cuprous oxide (rGO-Cu_2_O) have been prepared by the reduction of copper sulfate on graphene oxide for long-term antibacterial activities [94]. The P-type semiconductor of Cu_2_O may easily separate its electron-hole pairs under light condition. In this study, the fresh rGO-Cu_2_O nanocomposites generated more ROS compared to that of fresh rGO and Cu_2_O (Figure 11). The results indicated that the separation performance of photo-excited charges of Cu_2_O may be distinctly increased by the combination of Cu_2_O and rGO due to the improvement of interfacial charge transfer between Cu_2_O and rGO. The ROS antibacterial mechanism of rGO-Cu_2_O nanocomposites may be attributed to that the light-induced electrons were delivered from Cu_2_O to rGO to eliminate the recombination of the pairs of electrons and holes [95]. The photo-excited electrons and holes from Cu_2_O may be used for disinfection of bacteria [96]. Furthermore, rGO have played an important role for the acceptance of light-induced electrons from Cu_2_O to provide efficient charge transfer between the heterostructured nanocomposites of rGO-Cu_2_O and bacteria. The photo-excited electrons and holes may create intracellular ROS such as H_2_O_2_, ⋅OH and ⋅O_2_^−^. Eventually, the active substances of ROS could cause damage of nucleic acids, inactivation of intracellular protein, disability of the mitochondria and destruction of bacterial membrane.

**Figure 12:**
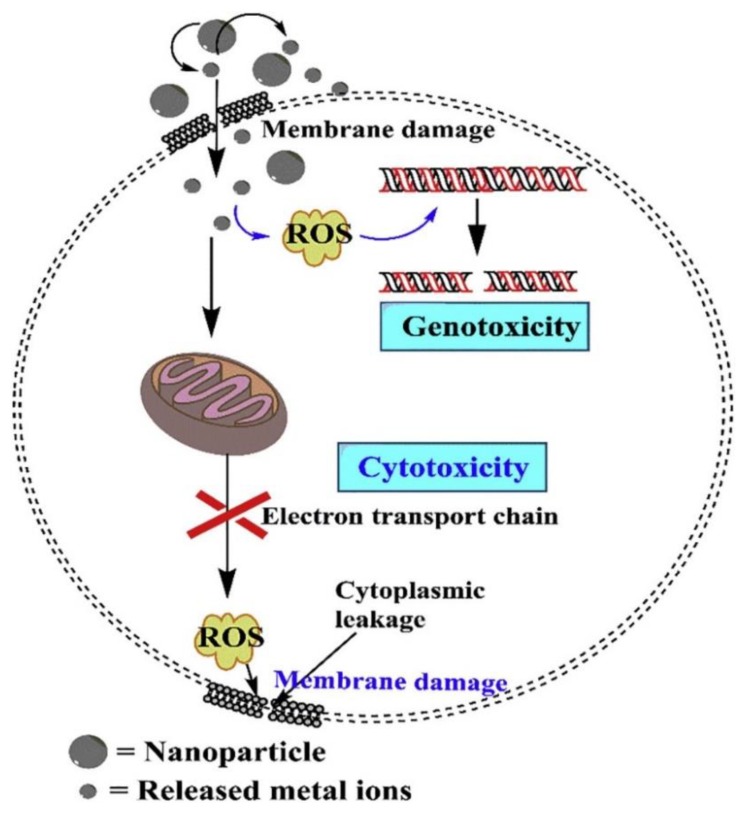
Antibacterial mechanisms of ZnO-Se nanocomposites with visible light illumination. Reproduced with permission from ref. [97]. Copyright © 2020, Elsevier.

Zinc oxide-selenium (ZnO-Se) heterojunction nanocomposites have been fabricated as antibacterial agents for *S. aureus* [97]. For the control, ZnO nanoparticles showed the zone of inhibition as 3.0 cm for *S. aureus* with visible light illumination. In the contrast, ZnO-Se nanocomposites revealed outstanding antibacterial activity with a zone of inhibition as 5 cm for *S. aureus* with visible light illumination. Furthermore, the bactericidal property of ZnO-Se nanocomposites remained for few days to inhibit the growth of *S. aureus*. The enhancement of antibacterial activity of ZnO-Se nanocomposites could be attributed to the increase of light-harvesting capability for sustainable production of ROS to kill *S. aureus* (Figure 12). To investigate the role of ROS in the antibacterial mechanism, electron spin resonance (ESR) was utilized to assess the types of ROS production from ZnO-Se nanocomposites. The results of ESR experiments demonstrated that ZnO-Se nanocomposite may produce ROS, including singlet oxygen and reactive OH species. Overall, the ZnO-Se heterojunction nanocomposites had long term enhancement of their antibacterial activity with visible light illumination and therefore, may be a potential antibacterial agent.

## 3. Challenges and Opportunities

In this review, we have compiled recent investigations of heterostructured nanomaterials as light-activated antibacterial agents in applications such as medicine, food safety, water sterilization and the textile industry (Table 1). These investigations have demonstrated that heterostructured nanomaterials may be high-performance antibacterial agents due to their synergistic effects under light irradiation. With synergistic effects, heterostructured nanomaterials exhibited excellent antibacterial activities based on the increase of ROS generation under light irradiation. Furthermore, the heterostructures of nanomaterials also inhibited the recombination of photon-induced electron/hole pairs for the increase of ROS formation to kill bacteria. Although various heterostructured nanomaterials have been shown to be light-activated antimicrobial agents, their antibacterial activities still need to be improved. For real clinical applications, the antibacterial efficiency of LAHNs should fit the requirement with a 4-log (99.99%) reduction in bacterial viability, which is one of the leading causes of nosocomial infections in the world. The first challenge to enhance the antimicrobial activity of LAHNs is to increase their synergistic effects. The development of LAHNs with novel compositions would be recommended. The second challenge to improve antibacterial activity for light-activated antimicrobial agents is to combine with other optical properties. For example, the photothermal effect is also used to kill bacteria with light irradiation. Therefore, with ROS generation and photothermal effects, LAHNs may provide better antimicrobial activities. The third challenge for light-activated antimicrobial agents is to increase their biocompatibility for the clinic application. To date, the cytotoxicity of LAHNs is still too high for clinic tests. However, in vitro and in vivo studies of light-activated antimicrobial agents of heterostructured nanomaterials are a critical step for future clinic application. Overall, to realize the antibacterial agents of LAHNs, great efforts are still needed for the improvement of their antibacterial activities. With intensive studies, we believe that antibacterial agents of LAHNs can be utilized as the important antibacterial agents in the near future.

## Figures and Tables

**Table 1:** Light-activated nanomaterials for antibacterial application in this review.

Nanomaterials	Light harvester	Light Source	Bacteria	References
Ag-TiO_2_	Ag	UV light	*B. subtilis* and *P. putida*	[68]
TiO_2_/AgVO_3_	AgVO_3_	visible light	*E. coli*	[75]
Ag/TiO_2_/cellulose	Ag	UV light	*E. coli*	[81]
Alginate/Au-TiO_2_	Au	visible light	*S. aureus* and *E. coli*	[82]
Li-TiO_2_/LDPE	Li	visible light	*S. aureus*	[83]
Cu-TiO_2_	Cu	visible light	*E. coli*	[84]
TiO_2_/α-Fe_2_O_3_	α-Fe_2_O_3_	visible light	*E. coli*	[85]
U,Bi-TiO_2_	N and Bi	visible light	*S. sanguinis* and *A. naeslundii*	[86]
CTS/MTiO_2_	melon	visible light	*S. aureus*	[87]
TiO_2_/GSs	TiO_2_	visible light	*E. coli*	[88]
rGO-Cu_2_O	Cu_2_O	sunlight	*S. aureus* and *E. coli*	[94]
ZnO-Se	Se	visible light	*S. aureus*	[97]
